# Governing access to medicines in Central America: community pharmacy, patient safety, and lessons from Spain

**DOI:** 10.3389/frhs.2026.1781501

**Published:** 2026-03-05

**Authors:** Esteban Zavaleta-Monestel, Luis Carlos Monge-Bogantes, Jeaustin Mora-Jiménez, Sebastián Arguedas-Chacón

**Affiliations:** 1 Research Department, Hospital Clínica Bíblica, San Jose, Costa Rica

**Keywords:** access to medicines, Central America, community pharmacy, health systems governance, minor ailment management, patient safety

## Introduction

In Central America, access to essential medicines is limited less by availability or affordability than by the governance of access points and treatment decision-making processes. Despite ongoing commitments to universal health coverage and investments in procurement and distribution, persistent access gaps and fragmented care pathways remain prevalent across the subregion. These challenges primarily reflect systemic weaknesses in service delivery design rather than shortages of pharmaceutical products (1–4).

Community pharmacies serve as a primary access point to healthcare throughout Central America. For many individuals, especially those in urban peripheries and rural areas, pharmacies are the most accessible and frequently utilized contact with the health system. Regional empirical evidence indicates that pharmaceutical indication or recommendations for minor ailments are among the most commonly requested services in private community pharmacies in Costa Rica, Guatemala, Honduras, Panama, and El Salvador. In this manuscript, the term pharmaceutical indication is used to describe pharmacist-led management of minor ailments, conceptually aligned with internationally recognized Minor Ailment Schemes or Common Conditions Services. However, the quality and regulatory support for these services vary considerably (5).

This paper presents a policy analysis focused on community pharmacy governance in Central America, specifically examining how regulatory design influences access, safety, and equity at the initial point of care. Rather than reevaluating pharmacists' clinical competence, which is well established in international literature, the analysis centers on the ways regulatory frameworks define, limit, or enable pharmacists' roles as formal access providers (6). Spain serves as a comparative reference, representing a mature European model where community pharmacy is legally integrated into primary care, pharmacist presence is mandatory, and pharmaceutical indication is recognized as a regulated professional service supported by protocols and referral pathways. This comparison underscores structural governance differences that help explain varying access outcomes in Central America (7).

### Access failures at the point of care

Foundational work on essential medicines has long emphasized that access failures are shaped not only by medicine lists, procurement, or pricing, but by how health systems organize service delivery and ensure appropriate use at the point of care (8). In low- and middle-income settings, barriers accumulate across financing, regulation, and workforce constraints, producing persistent inequities even when medicines are physically available (9).

Community pharmacies occupy a pivotal position in this access landscape. Evidence from diverse low-resource settings shows that pharmacies routinely function as first-contact providers for individuals seeking advice on symptoms, medication use, and self-care (10). However, weak regulatory environments often allow these clinical interactions to occur without standardized protocols or accountability, creating variability in safety and quality (11).

From a health systems perspective, true access to medicines is realized not simply through product availability at retail outlets, but when patients receive timely, appropriate, and safe treatment for their health needs. When community pharmacies operate as unregulated points of sale, access becomes nominal rather than functional, requiring patients to interpret symptoms, select therapies, and manage risks independently. In contrast, when pharmacies are governed as clinical access points, professional assessment at the time of medicine access serves as a risk-filtering mechanism, ensuring appropriate medicine selection, identification of contraindications, and referral when necessary. Strengthening governance at the point of care thus directly enhances both access and patient safety by transforming the availability of medicine into clinically supported treatment decisions.

When access to medicines occurs without professional guidance, the consequences are well documented. Unsupervised self-medication is associated with dosing errors, inappropriate medicine selection, harmful drug interactions, delayed diagnosis, and avoidable adverse outcomes (12). Prospective studies further link both over-the-counter and prescribed medicines used without adequate oversight to hospital admissions related to adverse drug reactions (13).

These risks are amplified in settings where pharmacies substitute for formal primary care. Evidence from informal settlements and rural communities illustrates both the centrality of pharmacies in care-seeking behavior and the vulnerabilities that arise when professional guidance is weak or unsupported (14).

Importantly, the harms associated with informal pharmacy-based access are not evenly distributed. Individuals with limited health literacy, chronic conditions, polypharmacy, or barriers to physician care are disproportionately exposed to risk when medicines are accessed without professional assessment. In these situations, weak governance effectively shifts responsibility for clinical decision-making onto patients, exacerbating inequities and increasing the likelihood of preventable adverse outcomes. Strengthening regulation of pharmaceutical indications, therefore, functions not only as a patient-safety intervention but also as an equity-oriented access reform.

### Central America: practice without governance

Recent empirical evidence confirms that pharmaceutical indication already functions as a de facto access pathway in Central America. A multi-country observational study of private community pharmacies found that pharmaceutical indications or recommendations for minor ailments are routinely provided across the region, yet frequently fail to meet internationally recognized standards for patient assessment, documentation, and follow-up (15).

The study shows considerable variation in pharmacist presence requirements, training, and regulatory enforcement across the region. Costa Rica exhibits stronger regulatory protections through mandatory on-site pharmacist presence, whereas other countries permit a single pharmacist to supervise multiple pharmacies or delegate patient care primarily to non-pharmacist staff. These findings suggest that access failures result not from insufficient professional activity, but from systematic under-regulation of services already being delivered.

### Regulatory design: Spain as a comparator

In Spain, community pharmacies operate under a tightly regulated legal framework that mandates the continuous physical presence of a licensed pharmacist and formally defines pharmaceutical indication as a professional service within primary care. Pharmacists are legally responsible for clinical assessment, counseling, and referral when symptoms exceed defined thresholds, supported by enforceable professional standards (16).

The Spanish model *suggests* that formal regulation of pharmaceutical indication can stabilize and legitimize access rather than restrict it. By establishing clear scopes of practice, documentation standards, and referral thresholds, governance frameworks appear to reduce uncertainty for both professionals and patients while maintaining rapid, community-level access to care. Regulation can enable accountability without sacrificing flexibility, allowing pharmacists to efficiently manage minor conditions and appropriately escalate more serious cases. From a governance perspective, this example can be interpreted as indicating that enhancing patient safety through governance may improve trust, consistency, and system responsiveness without necessarily compromising accessibility.

### Minor ailment management and public health functions

Evidence from European randomized trials demonstrates that structured pharmacist-led minor ailment services improve clinical outcomes, enhance appropriate referral, and reduce pressure on general practice without compromising safety (17). These findings are reinforced by patient preference studies and systematic reviews showing consistent benefits when pharmacy services are protocolized and integrated into care pathways (18).

Additional evidence from a Latin American healthcare network demonstrates that minor ailment consultations in pharmacies require significant clinical decision-making, and that regulatory compliance directly affects the appropriateness and safety of recommended therapies (19). This finding emphasizes that governance, rather than professional capacity, is the primary determinant of outcomes (19).

Pharmacist participation in public health services, particularly vaccination, provides a parallel example. International evidence shows that pharmacist-administered vaccination programs are safe, effective, and capable of expanding coverage when supported by legal authority and accountability mechanisms (20).

### Policy implications for Central American health systems

Enhancing governance of community pharmacy services in Central America presents an opportunity to both expand access to care and strengthen patient safety. Pharmaceutical indication and minor ailment management already serve as first-contact access pathways for much of the population; however, in the absence of formal governance, these pathways expose patients to preventable clinical risks. Policy reforms that regulate and support these services optimize existing access channels, ensuring that care is timely, clinically appropriate, and equitably delivered.

Four governance-level actions emerge as priorities. These actions are presented as complementary and interdependent policy measures rather than as a ranked or hierarchical sequence:

Mandatory on-site pharmacist presence during the provision of pharmaceutical indication and minor ailment services.
Formal recognition of pharmaceutical indication as a regulated professional service, supported by standardized protocols and documentation.
Remuneration for cognitive and clinical pharmacy services, decoupled from medicine sales, to align incentives with safe access.
Integration of community pharmacies into primary care and public health systems, including referral pathways and immunization strategies.

In the absence of these reforms, health systems will continue to depend on informal pharmacy-based access, thereby shifting clinical risk to patients instead of managing it within accountable frameworks.

## Conclusion

Spain's experience *suggests* that community pharmacies can serve as safe, effective, and equitable access points when supported by robust governance. In Central America, ongoing access failures are attributable not to insufficient professional capacity, but to the lack of formal recognition and regulation of the role pharmacies already fulfill at the front lines of care. Strengthening community pharmacy governance in this context is not an expansion of professional roles, but a pragmatic health systems intervention that ensures consistent access, safety, and equity at the most frequently utilized point of care.
